# Family doctors' knowledge and self-reported care of type 2 diabetes patients in comparison to the clinical practice guideline: cross-sectional study

**DOI:** 10.1186/1471-2296-7-36

**Published:** 2006-06-16

**Authors:** Anneli Rätsep, Ruth Kalda, Ivika Oja, Margus Lember

**Affiliations:** 1 Polyclinic and Family Medicine Department, University of Tartu, Tartu, Estonia; 2 General Practice of Laeva, Laeva, Tartu County, Estonia; 3 Department of Internal Medicine, University of Tartu, Tartu, Estonia

## Abstract

**Background:**

It is widely believed that providing doctors with guidelines will lead to more effective clinical practice and better patient care. However, different studies have shown contradictory results in quality improvement as a result of guideline implementation. The aim of this study was to compare family doctors' knowledge and self-reported care of type 2 diabetes patients with recommendation standards of the clinical practice guideline.

**Methods:**

In April 2003 a survey was conducted among family doctors in Estonia. The structured questionnaire focused on the knowledge and self-reported behavior of doctors regarding the guideline of type 2 diabetes. The demographic and professional data of the respondents was also provided.

**Results:**

Of the 354 questionnaires distributed, 163 were returned for a response rate of 46%. Seventy-six percent of the responded doctors stated that they had a copy of the guideline available while 24% reported that they did not. Eighty-three percent of the doctors considered it applicable and 79% reported using it in daily practice. The doctors tended to start treatment with medications and were satisfied with treatment outcomes at higher fasting blood glucose levels than the levels recommended in the guideline. Doctors' self-reported performance of the tests and examinations named in the guideline, which should be performed within a certain time limit, varied from overuse to underuse. Blood pressure, serum creatinine, eye examination and checking patients' ability to manage their diabetes were the best-followed items while glycosylated hemoglobin and weight reduction were the most poorly followed. Doctors' behavior was not related to the fact of whether they had the guideline available, whether they considered it applicable, or whether they actually used it.

**Conclusion:**

Doctors' knowledge and self-reported behavior in patient follow-up of type 2 diabetes is very variable and is not related to the reported availability or usage of the guideline. Practice guidelines may be a useful source of information but they should not be overestimated.

## Background

Clinical practice guidelines (CPG) are systematically developed statements to assist the decisions of the practitioner and patient about appropriate healthcare for specific clinical circumstances [1]. It is expected that clinical practice guidelines improve healthcare quality, reduce inappropriate variations between providers and predispose dissemination of the evidence-based medicine concept in daily practice. Policymakers and payers see guidelines as a tool for making healthcare more consistent and efficient. However, there is no certain evidence that guidelines may change practice behavior. Different studies have shown contradictory results in quality improvement as a result of guideline implementation. Some studies describe improvement in disease management after guideline dissemination [2-4] but evidence to the contrary exists that none of the guideline intervention strategies led to improvements in patient quality of life, quality of diabetes care or performance activity and adherence to the guideline [5-7]. The potential barriers to physicians' adherence to guidelines can be divided into three themes: physician knowledge, attitudes and behavior. Meanwhile the sequence of the "knowledge-attitude-behavior" model is important in modifying physicians' practice patterns [8].

In recent decades care of patients with diabetes type 2 (DM2) has shifted from specialist care to primary care [9,10]. The same trend has taken place in Estonia where in the 1990s previous highly specialized primary medical care was changed into a primary care-oriented and family doctor-based system [11]. To improve the quality of care, the Estonian Society of Family Doctors started to develop national practice guidelines in collaboration with specialist societies in 1994. The type 2 diabetes guidelines for family doctors (FD) are some of the latest, developed in 2000 by a multidisciplinary team, led by FDs and based on the International Diabetes Federation Europe DM2 guideline [12]. The guidelines was introduced to FDs at an educational seminar and disseminated by mail for all FD Society members.

Estonian family doctors' awareness of practice guidelines has not been assessed before. Hence our aim was to compare FDs' knowledge and self-reported care of type 2 diabetes patients with the recommendations of the clinical practice guideline.

## Methods

A questionnaire-based survey was conducted in 2003. Every second doctor (n = 354) from the list of the Estonian Society of Family Doctors received a questionnaire by mail. A second mailing with a reminder letter and an additional questionnaire were sent to those who had not responded three weeks after the initial mailing. The questionnaire had been compiled by a research team and had been piloted before using it in the study.

The questionnaire covered the following items:

### Background characteristics

Independent variables included the year of graduation from medical school, the year of specialization as a family doctor, practice type and location, practice size and the number of diabetes patients.

### Availability of the guideline

To the questions about the DM2 guideline availability, its use in daily practice and its estimated applicability, yes/no responses were required.

### Specialist accessibility

The doctors were asked about the possibility to consult an endocrinologist, the distance to the nearest endocrinologist and the opportunity to consult an endocrinologist by telephone.

### Following the guideline

The doctors were asked to report the level of blood glucose at which they usually start treatment with medications if lifestyle changes have been ineffective, and the level at which they are content with treatment outcome. In the DM2 guideline, HbAc1 is suggested for assessment of glucose control and corresponding target levels of capillary plasma glucose levels are provided. In the current study the doctors were asked to provide the respective fasting capillary plasma glucose levels, as this analysis was more widely used by the doctors at the time of study performance. The suggested level for starting treatment with medication is >6.5% for HbAc1 and >6.1 mmol/L (109.8 mg/dl) for capillary plasma glucose.

In the next section the family doctors were asked about the frequency at which they perform the following tests and examinations prescribed in the guideline: checking symptoms/complications, checking the patients' ability to manage their diabetes, smoking habit, blood pressure, weight/BMI, foot exam, eye exam, HbAc1, lipids (LDL, HDL, and TG), urinary protein, urinary albumin, and serum creatinine. The response options were "once a month," "at least once every three months," "at least annually," "rarely," and "I do not consider it necessary."

In the Estonian type 2 diabetes guideline, checking the patients' ability to manage their diabetes and the blood pressure measurement is suggested to be performed at every visit. In case of the latter, the doctors' responses "every visit" and "at least once every three months"in the questionnaire were deemed appropriate according to the guideline. HbAc1 is recommended to be checked every three months and other items annually.

Score for adherence to the guideline was calculated for each physician depending on how many guideline recommendations of 12 test and examinations have been timely performed according to their self-assessment report. More frequent performance was assessed as non-adherence as unneeded use of resources.

### Statistical analysis

The obtained responses were entered in a database and were analysed by SPSS 10.0 (Statistical Package for the Social Sciences) for Windows. Statistical analysis included the chi-square test for the categorical variables and analysis of variance for the mean continuous variables. All calculated p-values were two-tailed. The P-values higher than 0.05 were considered insignificant.

## Results

Of the 354 doctors who received the questionnaire 46% (n = 163) responded. There were no significant differences between respondents in the first and second mailings. The background characteristics of the respondents in our sample correspond to that of the members of the Estonian Family Medicine Society, except for working area. Representation of doctors from cities was lower among respondents (19% vs. 37%) but no difference was found in any comparison between urban and rural doctors.

### Background characteristics and availability of the guideline

The mean size of the patient list was 1830 ± 407 and the average (± SD) working experience 22 ± 7.0 years. Regarding their previous specialty, the majority of the respondents had been district doctors for adults. Fifty-three percent of the doctors worked in solo practices and the rest worked in group practices (Table 1).

**Table 1:** Background characteristics of the primary care providers according to the previous specialty, type of practice and working area

Criterion	n	%
Status before specialization as a family physician		
District doctor for adults	109	67
District pediatrician	36	22
Other specialties	9	6
Family physician through residency	8	5
		
Type of practice		
Solo	85	53
Group	76	47
		
Working area		
Urban	67	42
Rural	63	39
City	30	19

The median number of diabetes patients in the list was 35.

Seventy-six percent of the respondents stated that the guideline was available while 24% reported that it was not. Eighty-three percent of the doctors considered it applicable and 79% reported using it in daily practice. The availability and use of the guideline were not related to working area, practice type and size, previous status before specialization as an FD, waiting time or distance to an endocrinologist.

### Treatment decision and treatment goals

**Figure 1:**
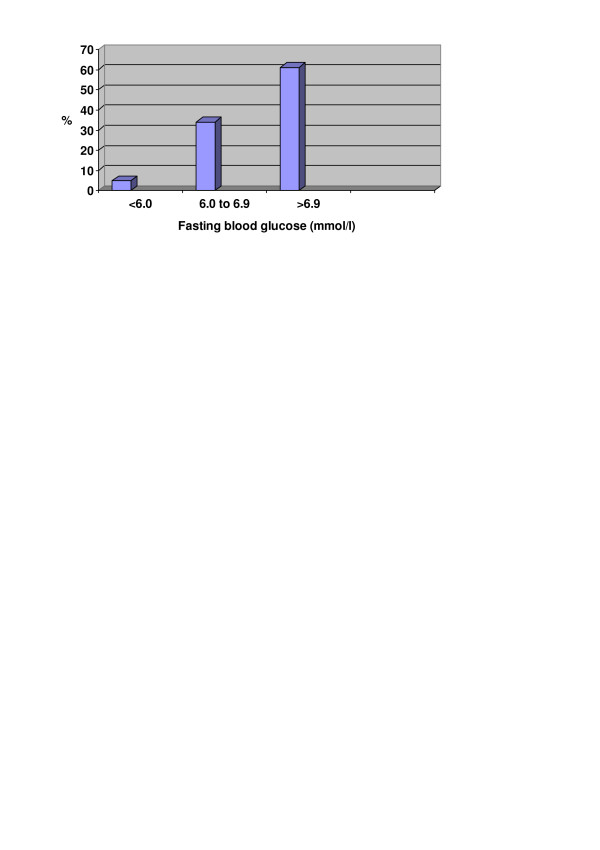
Distribution of doctors according to their decision to start treatment with medications depending on fasting blood glucose level.

On average, the doctors tended to start treatment with medications at higher fasting blood glucose (FBG) levels than the levels recommended in the guideline (Table 2). More than half of the doctors made a decision to start treatment with medications on FBG above 7 mmol/l, while a few made this decision at FBG values below 6.1 mmol/l (Figure 1). The decision about when to start treatment with medications was not related to whether the doctors had the guideline available, whether they considered it applicable, or whether they actually used it. There were no differences in the treatment behavior depending on the number of patients with diabetes in the doctors' list.

**Table 2:** Fasting blood glucose values (mmol/l) at which a decision to start treatment with medications was taken and the values at which doctors were content with treatment outcome

	Mean (SD)	Minimum	Maximum	Standard in DM guideline
Decision to start treatment with medication	7.2 (1.3)	5.5	15.0	6.1
Satisfaction with treatment outcome (glycemic control)	6.8 (1.4)	5.0	14.0	5.5

### Following the guideline

**Figure 2:**
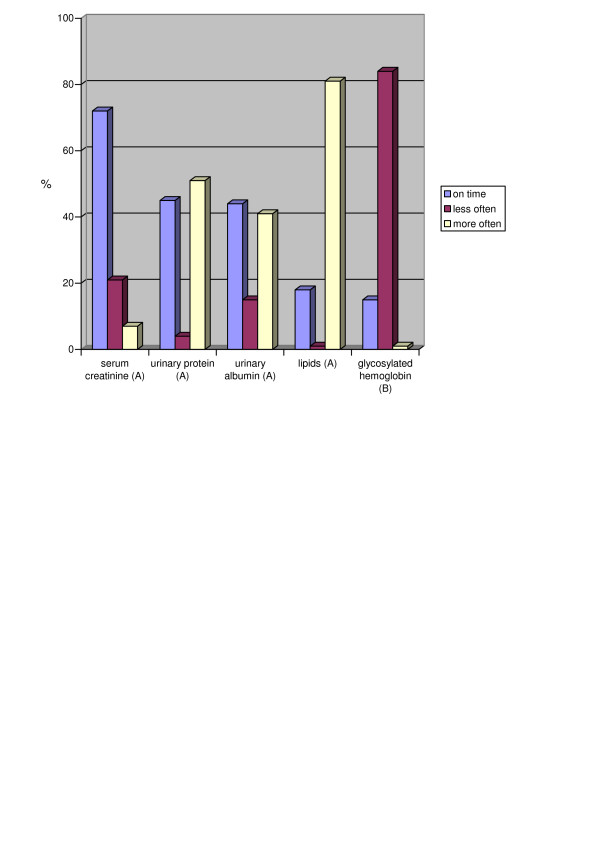
**Proportions of the doctors and their variation in performing the tests recommended in the guideline**. Recommended frequency in the CPG: A – annually, B – every 3 months

**Figure 3:**
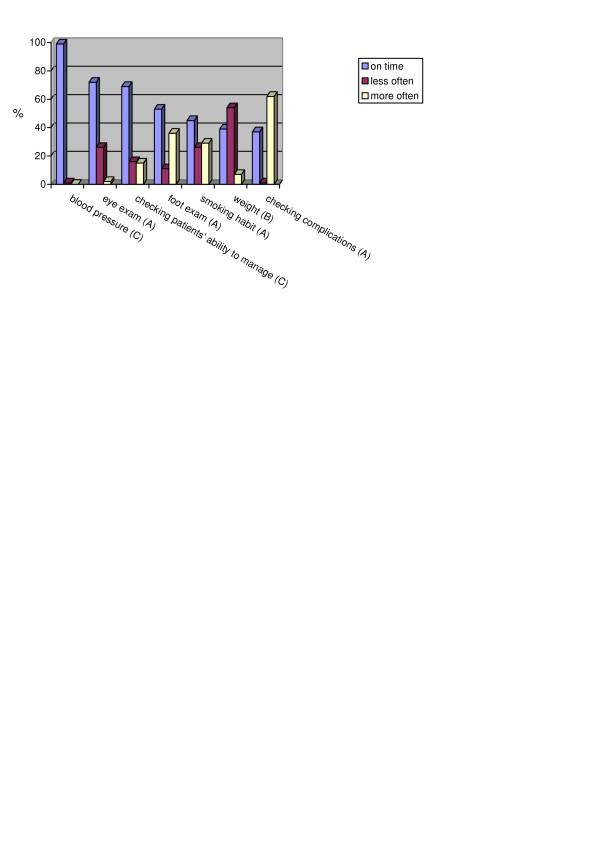
**Proportions of doctors and their variations in performing the exams recommended in the guideline**. Recommended frequency in the CPG: A – annually, B – every 3 months, C – every visit

The DM2 guideline includes 12 tests and examinations which should be performed during the year. According to the self-reported performance of the tests and examinations, it varied from overuse to underuse (Figures 2, 3).

Blood pressure, serum creatinine, eye examination and checking patients' ability to manage their diabetes were the best followed tests and examinations while glycosylated hemoglobin and weight reduction were the most poorly followed. Checking symptoms and complications as well as checking urinary protein and albumin were performed more often than recommended in the guideline.

According to the score of adherence none of the family doctors performed all the required tests and examinations on time (Table 3). At least half of the tests and examinations listed in the guideline were performed on time by 52% of the family doctors. Maximum score 12, was not reached by any of the doctors. The respondents' behavior in performing the tests and examinations did not depend on whether they had the guideline available or whether they had used it. There also was no difference in the doctors' behavior regarding the reported adherence to the guideline in terms of background characteristics and specialist accessibility.

**Table 3:** Distribution of the doctors according to the adherence to the DM2 guideline (adherence score)

Adherence score	Number of doctors	Cumulative %
12	0	0
11	0	0
10	1	1
9	6	5
8	19	26
7	27	32
6	33	52
5	29	70
4	26	86
3	12	93
2	6	97
1	1	98
None	3	100
TOTAL	163	100

## Discussion

The present study assessed whether FDs were aware of and familiar with the recommendations in the DM2 guideline and whether this was related to FDs' previous specialty, background characteristics or specialist accessibility. This is the first time CPG application has been studied in Estonia.

CPGs have been developed and used in the US for more than three decades and about 1500 guidelines are available for the American Medical Association. This is a large number of guidelines to expect to be followed. A recent trend in Estonia is to facilitate the development of different guidelines. At present, about 20 guidelines have been compiled for family doctors. However, as the number of guidelines is increasing, their usage as well as the actual awareness of them may decline and expected improvement in the quality of care is questionable.

According to the present study, almost two-thirds of the family doctors have the DM2 guideline at their disposal, and the majority of the doctors consider them applicable and use it in daily practice. This is comparable to the results of other studies demonstrating a positive attitude towards CPGs. Eighty-three percent of Israeli family physicians believed the guideline could be implemented and 75% attained help in the management of patients with DM [13]. Those findings are significantly different from several other studies where guidelines are available for only about one-fourth or less of family physicians, while even fewer still report using them [14,15]. The reason for this may be that doctors are sometimes doubtful about CPGs, and they consider them less useful than other sources of medical information, as they are developed for reducing healthcare costs and may not be applicable for individual patients and for local settings [16,17]. The usage of the guideline and the knowledge about it may also depend on how CPGs are distributed and if special educational activities are undertaken. However, the results of this study did not reveal any difference in the provision of diabetes care between the doctors who had CPGs available and used it in comparison with those who did not.

Regarding general factors that might influence usage of CPGs, some studies have shown that younger doctors considered CPGs more useful than older doctors [18] but there was no difference in our study.

### Decision of treatment

The results of the current study demonstrate that FDs mostly start treatment at higher FBG levels and the number of patients whom they considered to be compensated is low. The doctors who had guidelines and used them reported acting in the same way as those who did not. This shows that even if FDs had the relevant knowledge, many of them were reluctant to use it. On the other hand, the knowledge that they use may not have been derived from CPGs but from other sources.

The overwhelming majority of the FDs in our study tended to start treatment and were content with treatment outcome at higher levels of FBG than recommended in the guideline. It has been similarly reported that Italian physicians are content with treatment outcome at quite high FBG levels [19]. Other studies support the idea that doctors are not fully aware of the recommended criteria of CPGs for intensive blood glucose treatment [19,20]. Characteristics such as practice location, practice type, list size and length of experience in our study did not predict the glycemic control of type 2 diabetic patients, which coincides with the finding of another study [21].

### Following the guideline

Despite the fact that the majority of the doctors reported using the guideline, their knowledge of the tests and examinations recommended in the guideline were very variable. Blood pressure measurement was followed best, which is consistent with the findings from another study [22]. Of the laboratory tests, the performance of creatinine showed the best concordance with the guideline. In a similar American study, the performance of creatinine, proteinuria and HbAc1 tests was higher than 90% and had increased compared with the early nineties [22].

There were significant differences between the performances of the tests, from underuse to overuse, which might indicate the preferences of individual doctors. A factor leading to underuse of laboratory tests might be a lack of resources, which depends on the healthcare system and the financial system [11].

The results of this study showed that regardless of whether the doctors had the guideline available or not, the process of diabetes care remained very variable. It can be presumed that guidelines are not the only source for acquiring knowledge and information.

Following the guideline might be influenced by the guideline's dissemination and implementation strategy. It is evident that a part of Estonian FDs do not have a copy of the guideline available thus the process of translating diabetes guidelines into practice has occurred by diffusion [23] and partial dissemination [23]. Nevertheless there is imperfect evidence to support decisions about which guideline dissemination and implementation strategies are likely to be efficient under different circumstances [24].

However, guidelines cannot address all uncertainties in current clinical practice and should only be seen as one strategy among others that can help improve the quality of care that patients receive.

### Limitations of the study

Despite the low response rate, the results can still be generalized as the final structure of the respondents and non-respondents did not differ from each other. It is quite possible that self-reported data overestimates the real behavior of the doctors. Hereby according to our data there may be even more cause for concern for provided care. Even with these limitations the present study provides valuable information about the knowledge and behavior of FDs, who most frequently provide care for DM2 patients in Estonia.

## Conclusion

Guidelines are widely available, and are perceived as a useful and helpful source by most practitioners. Nevertheless, the way FDs take care of patients with diabetes varies remarkably. Updated and evidence-based guidelines can be useful as an educational tool but still a knowledge gap and variable behavior in clinical performance exist.

## Authors' contributions

ML, RK and AR participated in the design of the survey. RK, AR and IO carried out the study. ML, RK and AR contributed to the statistical analyses of the work. AR drafted the manuscript. AR, RK, IO and ML participated in the critical revisions of the manuscript. All authors read and approved the final manuscript.

## Pre-publication history

The pre-publication history for this paper can be accessed here:


